# Examining Barriers and Strengthening Community Collaboration as a Means to Increase Exclusive Breastfeeding Rates in Sarasota County, Florida

**DOI:** 10.7759/cureus.45022

**Published:** 2023-09-11

**Authors:** Kayla Rykiel, Julian Melchor, Brittany Long, Marc Chartier, Cynthia Samra

**Affiliations:** 1 College of Medicine, Florida State University College of Medicine, Tallahassee, USA; 2 College of Medicine, Florida State University College of Medicine, Sarasota, USA

**Keywords:** healthcare quality improvement, community medicine, community agencies, quality improvement tool, lactation consultant, breastfeeding duration, breastfeeding education, breastfeeding woman

## Abstract

Background

Maternal and infant benefits of breastfeeding have been established and widely recommended by the American Academy of Pediatrics. However, there is a discrepancy between the number of mothers initiating breastfeeding and those continuing to breastfeed for six months and beyond. In this project, we investigated current breastfeeding practices and barriers to feeding for mothers in Sarasota County, Florida to develop an intervention aimed at increasing the rate of exclusive breastfeeding at six months and beyond.

Methodology

The PDSA framework was used to develop interventions aimed at increasing breastfeeding rates in Sarasota County. Initially, breastfeeding mothers were administered a survey to understand breastfeeding practices and barriers. Community agencies that supported breastfeeding practices were identified. Based on survey data and feedback, a community breastfeeding conference was organized to improve collaboration and increase breastfeeding rates. Attendees’ knowledge and confidence were assessed through a conference pre-test and post-test. Additionally, attendees developed Smart, Measurable, Achievable, Relevant, and Timely goals that were recorded for data collection.

Results

Of the 28 completed community breastfeeding surveys, the respondents were of Caucasian or Hispanic ethnicity with an average age of 31. The majority of respondents had a goal of breastfeeding for 12 months, but only two participants reported that they continued to exclusively breastfeed to the 12-month mark. A total of 38 individuals from different agencies pre-registered for the conference; of these pre-registrants, 19 individuals checked in for conference attendance, 17 completed the conference pre-test, and 15 completed the conference post-test. Each domain surveyed demonstrated an increase in the values.

Conclusions

Following our intervention, there is an evident need for improvement in the pipeline of expanding collaboration among breastfeeding community agencies in Sarasota County. Upon completing our community breastfeeding conference, it was shown that this intervention provided both an educational improvement (demonstrated by increased mean domain scores) and a novel platform for providers to network. Our project highlights that strengthening the existing breastfeeding service infrastructure may directly increase exclusive breastfeeding rates at six months and beyond. Future interventions will aim to solidify recurrent infrastructural processes and policies.

## Introduction

It is well established that breastfeeding has many health benefits for both the mother and infant. The formal recommendation by the American Academy of Pediatrics (AAP) is to exclusively breastfeed for the first six months of life. After six months, the AAP recommends continued breastfeeding as additional foods are introduced into the child’s diet until two years or beyond [1]. This recommendation is widely supported and promoted by various other organizations including the American College of Obstetrics and Gynecology and the World Health Organization [2,3]. Some health benefits of breastfeeding for the infant include decreased rates of respiratory tract infections, severe diarrhea, ear infections, obesity, and a lower risk of sudden infant death syndrome [1]. Much of the immune protection in the breastfed child is attributed to the antimicrobial, anti-inflammatory, and immunoregulatory components that exist in human breast milk [3].

For the mother, breastfeeding is associated with both short-term and long-term health benefits. The short-term benefits include decreased postpartum blood loss and a lower risk of maternal anemia [1,3]. There are cardiovascular benefits in the short term of higher high-density lipoprotein (HDL) levels and lower postpartum cholesterol. For long-term benefits, studies have shown an inverse relationship between the duration of breastfeeding and the risk of development of postpartum chronic conditions, including hypertension, diabetes mellitus, cardiovascular disease, and stroke [3]. For example, the Women’s Health Initiative (WHI) study showed that breastfeeding for more than 12 months was associated with a 22% reduction in postmenopausal hypertension [3]. Breastfeeding has also been shown to have a reduction in cancer risk [1,3]. The WHI study showed that breastfeeding, regardless of the length of time, showed a 30% reduction in the risk of ovarian cancer and a 22% reduction in the risk of breast cancer compared to women who never breastfed [3].

Healthy People 2030 is a set of national objectives aimed at improving the health and well-being of the US population over the next decade. One of the mainstay goals of Healthy People 2030 is to increase the number of infants who are breastfed [4]. In the United States, it is reported that only 25% of women breastfeed exclusively through the first six months and 35% breastfeed through the first year [5]. To reach the target goal of 42.4% of infants who are breastfed exclusively through the age of six months, the state of Florida needs to increase exclusive breastfeeding by 24% [5]. Additionally, to reach the target goal of 50% for breastfeeding in one year, Florida needs to increase breastfeeding by 31% [5].

These data points highlight the substantial gaps in sustaining breastfeeding goals that remain despite the United States having high rates of breastfeeding initiation. It has been well-established that racial and socioeconomic barriers impact breastfeeding rates [6]. For example, women who participate in the Supplemental Nutrition Program for Women, Infants, and Children (WIC) have been shown to have lower breastfeeding rates. Socioeconomic demographics within the WIC program show further differentiation with lower-income women having a breastfeeding rate of 67.5% compared to higher-income WIC participants having a rate of 84.6% [6].

Because premature discontinued breastfeeding disproportionately affects minority women, it is particularly important to investigate the reasons why. There are many frequently cited barriers to breastfeeding that have been established through the literature, including cultural barriers, lack of information, access to and convenience of formula, and difficulty maintaining breastfeeding practices once women return to work [3,6].

Sarasota, Florida is a very resource-rich location. A recent report notes that there are nearly 2,500 established non-profits in the area [7]. For perspective, there are 48 non-profits for every 10,000 residents [7]. Therefore, it is uniquely important in Sarasota to further understand barriers to breastfeeding that might differ from those commonly cited in the literature. Given the resources, it is not a question of quantity but rather evaluating the quality and methods of delivery.

In 2021, approximately 90% of mothers in Sarasota County initiated breastfeeding at the time of birth. However, according to a Maternal Child Health Survey conducted in partnership with the Healthy Start Coalition of Sarasota County, only 20 out of 100 mothers surveyed reported breastfeeding for more than six months [8]. Despite this data being limited, it identifies a disconnect between the number of mothers who initiate breastfeeding versus the number of mothers who continue to breastfeed.

The quality improvement project described in this article was conducted to investigate breastfeeding practices and the barriers to exclusive breastfeeding among mothers in Sarasota County, Florida to develop interventions aimed at increasing the number of breastfeeding mothers, especially at six months and beyond.

## Materials and methods

**Figure 1:**
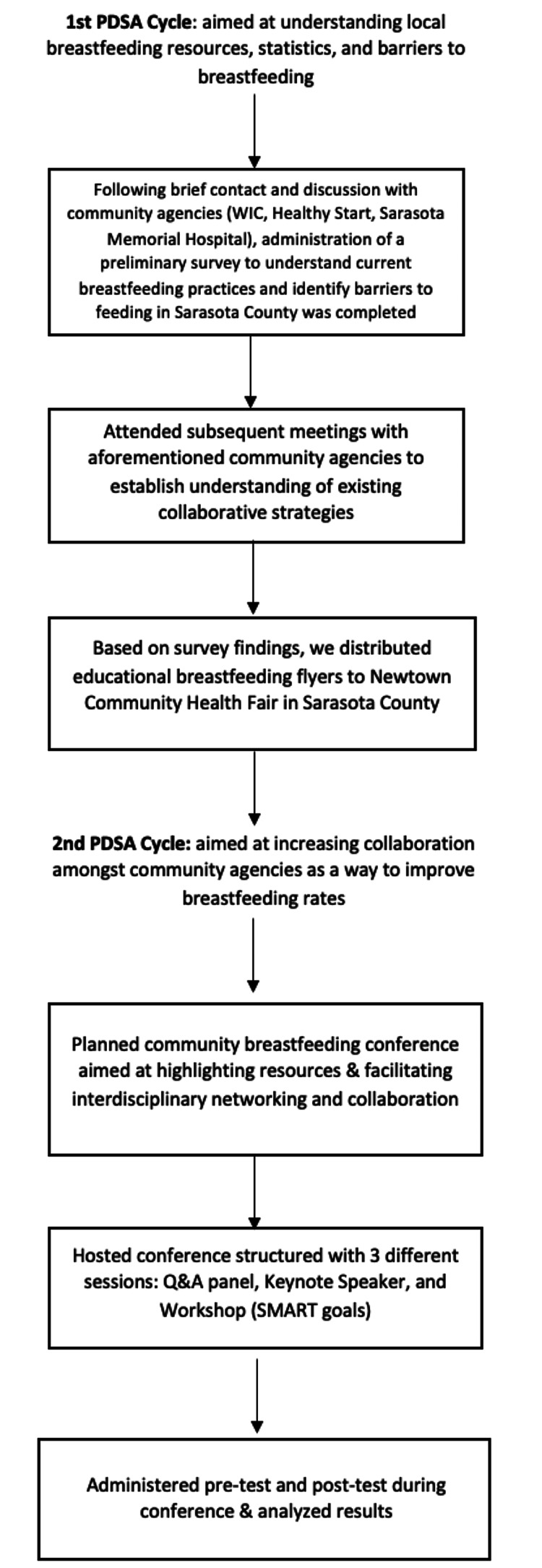
Flowchart displaying the project’s two Plan, Do, Study, Act (PDSA) cycle evolutions.

This quality improvement project was conducted by four third-year medical students from Florida State University under the supervision of the Quality Control Administrator of the Florida Department of Health-Sarasota County (FDOH-Sarasota County). The project focused on breastfeeding mothers currently engaged with community breastfeeding agencies in Sarasota County, Florida, and close collaboration with other healthcare organizations that provided support and resources to that population. The Plan-Do-Study-Act (PDSA) framework was employed throughout this project (Figure 1).

In the initial phase of this project, our group had individual meetings with representatives from Healthy Start, the WIC Nutrition Program, and the Women and Children’s Services at Sarasota Memorial Health Care System (SMHCS). Healthy Start and WIC exist as sub-organizations through the FDOH-Sarasota County. These meetings served as an opportunity to learn about the services that each organization provides.

**Figure 2:**
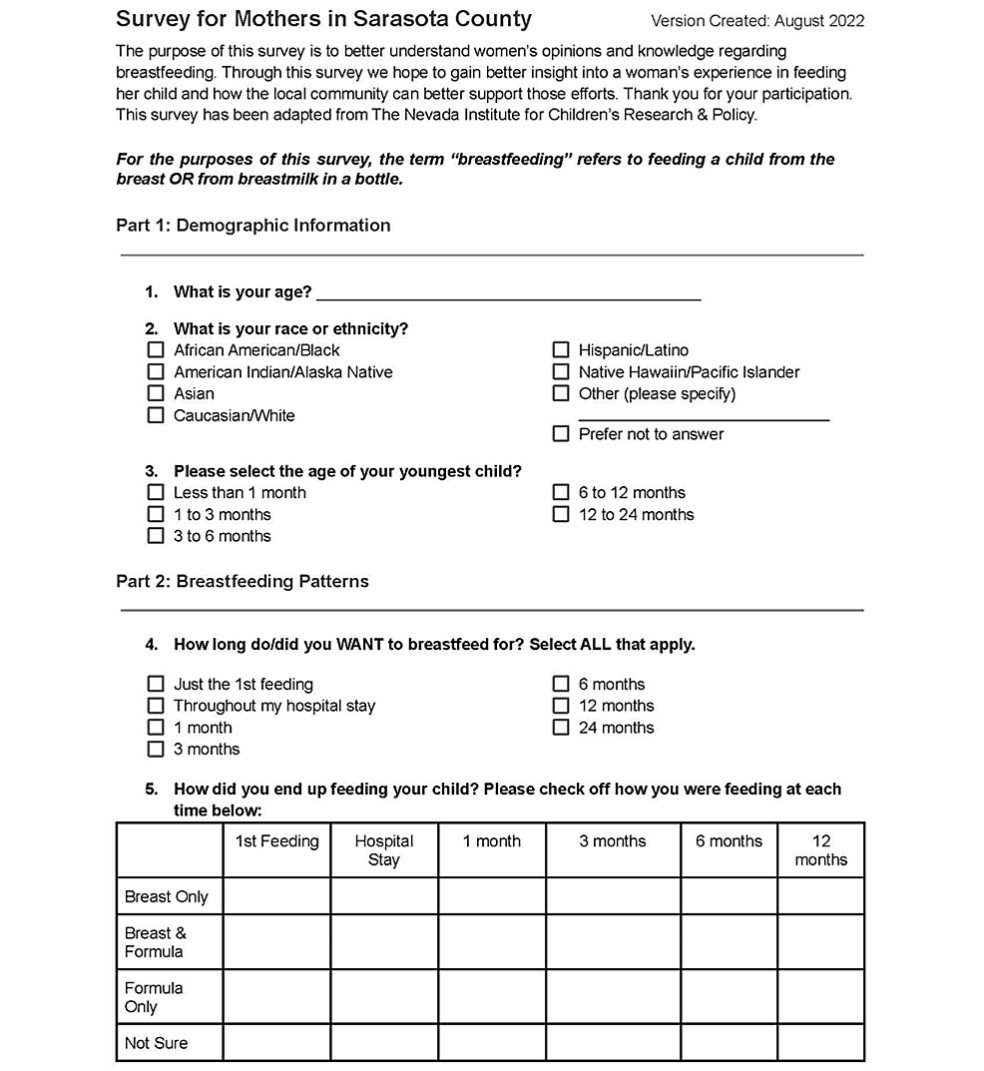
Questions 1-5 of the Maternal Breastfeeding Community Survey administered to mothers participating in breastfeeding services provided by Healthy Start and Women, Infants, and Children Nutrition Program.

**Figure 3:**
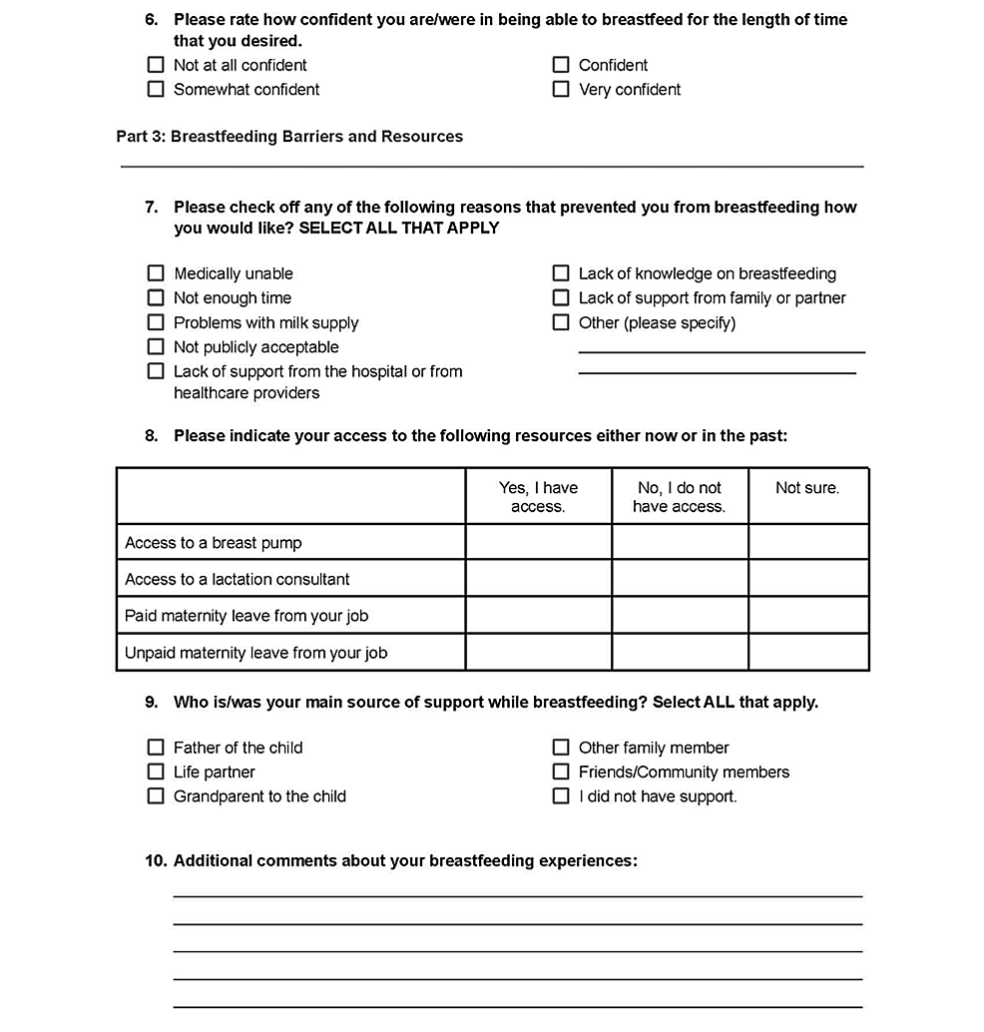
Questions 6-10 of the Maternal Breastfeeding Community Survey administered to mothers participating in breastfeeding services provided by Healthy Start and Women, Infants, and Children Nutrition Program.

Our team contributed to the creation of a Maternal Breastfeeding Community Survey in collaboration with the administration from Healthy Start and WIC (Figures 2, 3) to elicit region-specific barriers to prolonged breastfeeding. Lactation specialists and nutrition consultants from both organizations subsequently surveyed their own clientele. They periodically collected data which was then provided to our team for review and analysis from November 2022 to December 2022. A total of 28 surveys were collected throughout the PDSA cycle.

In early January of 2023, the group preliminarily evaluated our entire findings from the community breastfeeding survey and determined that one limiting factor to women’s success in breastfeeding was a lack of education about community resources. In an attempt to improve this, our group created and distributed educational flyers at the 2023 Newtown Community Screenings and Health Fair (Appendix 1). Newtown is a historically African American community located near downtown Sarasota. The fair was developed to encourage health participation in a predominantly low-income minority community. We distributed 50 flyers in total and provided some educational support to residents through conversation. The feedback we received when talking to community members was that they still felt there was difficulty in knowing and accessing breastfeeding services. This concluded our first PDSA cycle.

After the completion of our first PDSA cycle, our team reflected on our progress and felt it would be appropriate to develop a second PDSA cycle more specifically aimed at increasing collaboration among community agencies as a way to improve breastfeeding rates. Through meetings with Healthy Start, WIC, and SMHCS, we identified that there was an interruption in breastfeeding practices that occurred mostly during the transition from hospital services to community-based services. Therefore, we felt that our efforts would best be utilized by specifically targeting the translation of services and providing an opportunity for better collaboration through the development of an annual community conference.

From January 2023 through February 2023, our team organized the structure of the conference using the feedback we received during our first PDSA cycle. The conference advertising was aimed at individuals and organizations in the Sarasota area who were involved in supporting breastfeeding practices. This included non-profits, hospitals, the local health department, pediatricians, and obstetrician-gynecologists. The conference was advertised to over 40 organizations via email, calls, and in-person visits by members of the project with a flyer (Appendix 2) and a registration link through the website Eventbrite.

Our team structured and planned the community breastfeeding conference to serve as a platform to discuss current resources and facilitate interdisciplinary networking and collaboration among community partners. We developed a preliminary agenda and identified appropriate speakers for the conference. Speakers were chosen due to their experience within the field, position within the organization, and willingness to present at the conference. The agenda consisted of three sessions: a question and answer panel, a keynote speech, and a workshop (Appendix 3). The question and answer panel was entitled “What breastfeeding education looks like in the community.” The panel consisted of the Senior Human Program Services Specialist and certified lactation counselor from Healthy Start, the director of the WIC Nutrition Program, and the Community Outreach Manager from the Women and Children’s Services at SMHCS. The keynote speech was given by the Executive Director of the Women and Children’s Services at SMHCS on the topic of creating successful community partnerships. Lastly, the workshop was an opportunity for attendees to set future goals using the Specific, Measurable, Achievable, Realistic, and Timely (S.M.A.R.T.) goal framework (Appendix 4) [9]. The created goals were collected at the end of the workshop.

Data analysis

**Figure 4:**
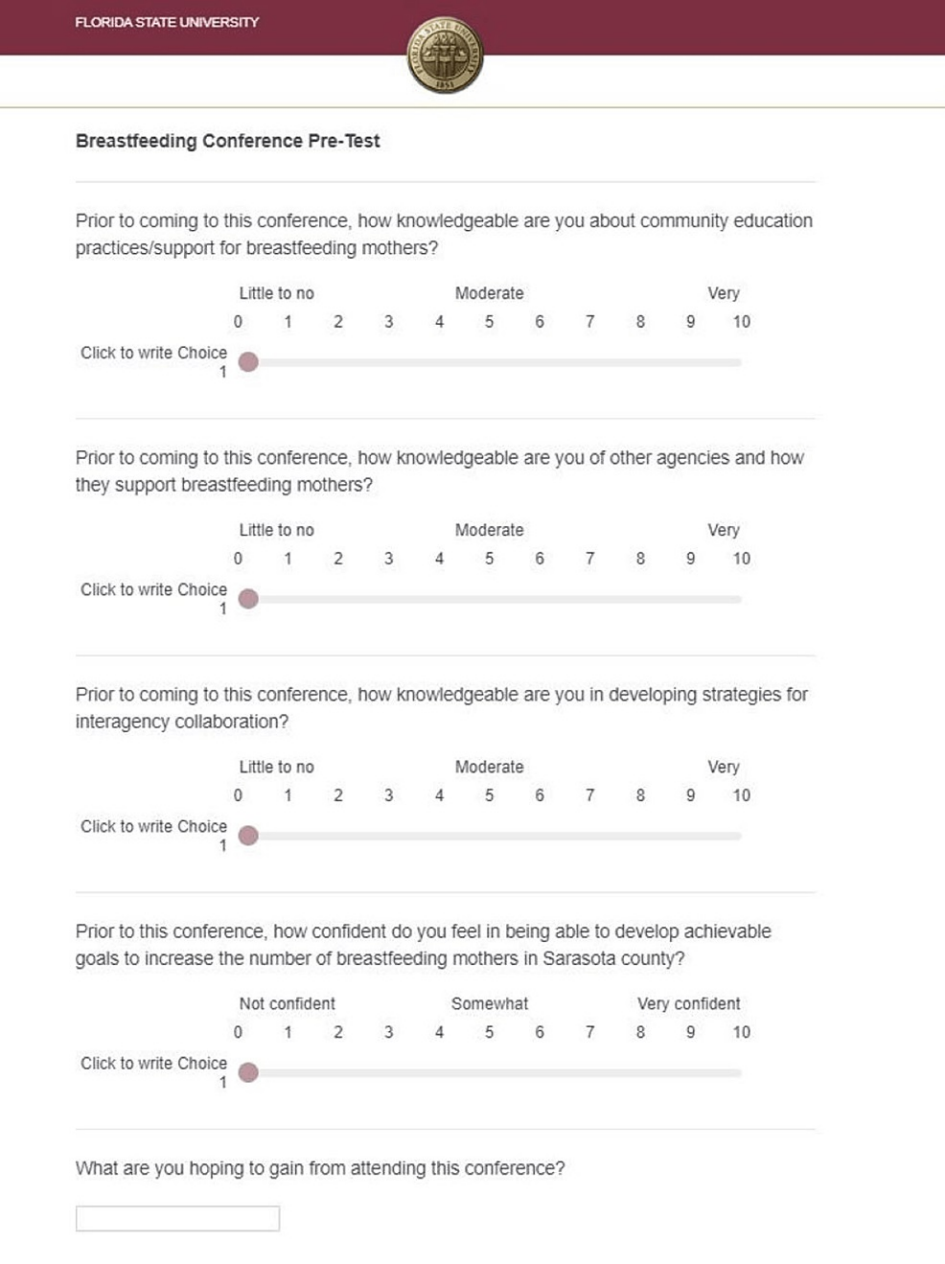
Conference Pre-Test for attendees of the Inaugural Sarasota Community Breastfeeding Conference.

**Figure 5:**
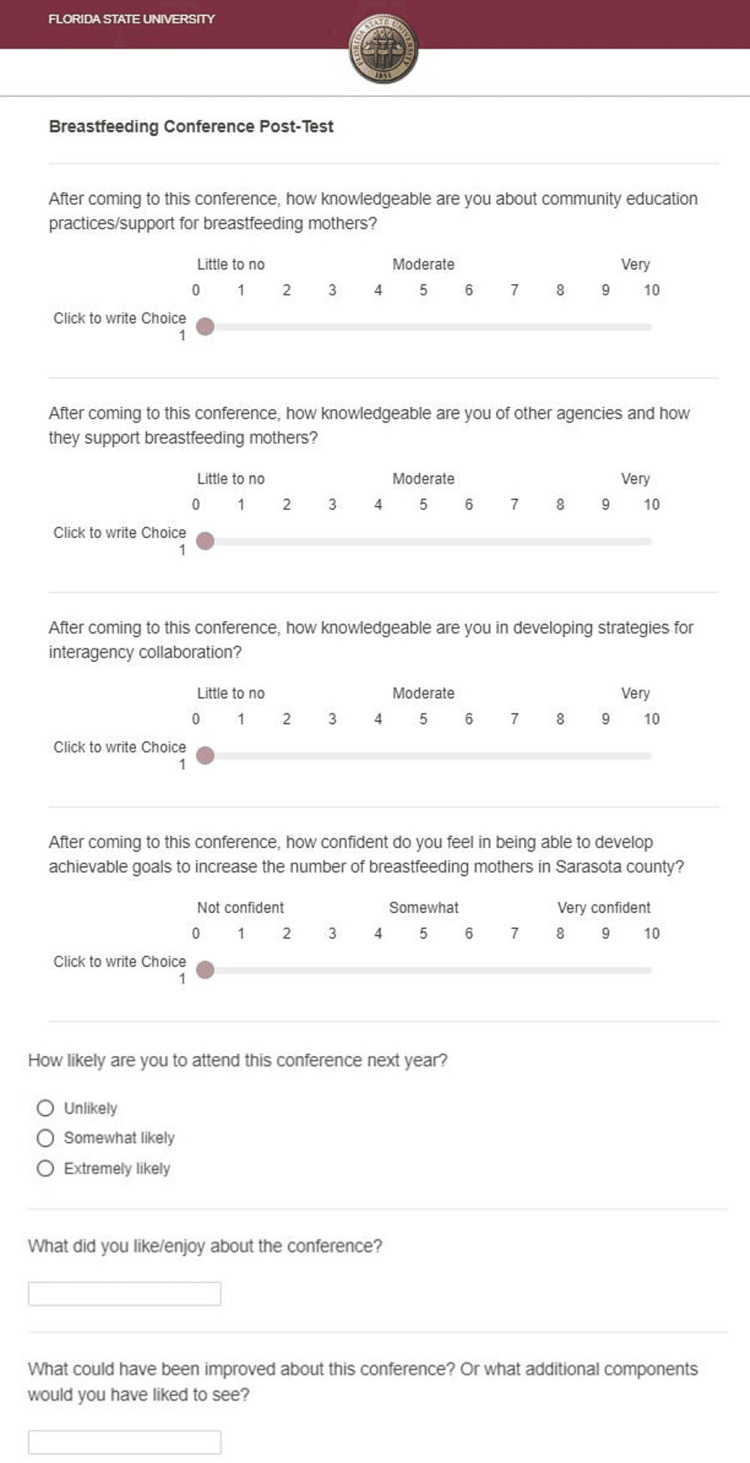
Conference post-test for attendees of the Inaugural Sarasota Community Breastfeeding Conference.

We performed a qualitative and quantitative data analysis of the community maternal breastfeeding surveys. During the conference, a pre-test and post-test were administered to elicit attendees’ perceived knowledge level on a scale of 0-10 regarding the following: community education practices/support for breastfeeding mothers, community agencies and how they support breastfeeding mothers, and lastly, developing strategies for interagency collaboration. Open-ended feedback was also obtained to better understand the perceived strengths and weaknesses of the conference. The pre-test included five questions and the post-test included seven total questions (Figures 4, 5). Responses were collected through the Qualtrics electronic survey platform which provided the minimum, maximum, and mean scores of each scaled question.

## Results

The questions and responses to the Maternal Breastfeeding Community Survey during the first PDSA cycle are summarized in Table 1. There was a total of 28 completed surveys. The average age of participants was 31 years old, with 12 respondents identifying as Caucasian, 10 identifying as Hispanic, and four identifying as African American/Black. The majority of respondents had the goal of breastfeeding for 12 months. Of the 22 participants who attempted exclusive breastfeeding, only six mothers reported exclusive breastfeeding at six months and two mothers at 12 months. Other participants moved to a combination of breastfeeding and formula feeds with four mothers doing a combination at six months and two mothers at 12 months. Most participants claimed to feel very confident in their knowledge of breastfeeding (11), with nine other mothers describing their knowledge as “confident” and five mothers as “somewhat confident.” None of the respondents were not at all confident. Problems with milk supply seemed to be the most common barrier experienced by participants, with almost half of the participants not provided paid maternity leave from their occupations. Support from the fathers was the most reported main source of support during breastfeeding, with grandparents and friends/community partners being other common support systems for breastfeeding mothers.

**Table 1:** Responses to the Maternal Breastfeeding Community Survey.

Questions (n = 10)	Answers from mothers in the Sarasota Community (n = 28)
1. What is your age?	
Average	31 (23–41) years
2. What is your race or ethnicity?	
African American/Black	4
Caucasian/White	12
Hispanic/Latino	10
3. Please select the age of your first child	
Less than 1 month	5
1 to 3 months	9
3 to 6 months	8
6 to 12 months	2
12 to 24 months	2
4. How long do/did you WANT to breastfeed for? Select ALL that apply	
1 month	2
3 months	1
6 months	5
12 months	13
24 months	5
5. How long did you end up feeding your child? Please check off how you were feeding at each time below	
Number of mothers feeding via breast only	
1^st^ feeding	22
Hospital stay	20
1 month	15
3 months	9
6 months	6
12 months	2
Number of mothers feeding via breast and formula	
1^st^ feeding	3
Hospital stay	6
1 month	9
3 months	9
6 months	4
12 months	2
6. Please rate how confident you are/were in being able to breastfeed for the length of time that you desired	
Somewhat confident	5
Confident	9
Very confident	11
7. Please check off any of the following reasons that prevented you from breastfeeding how you would like. SELECT ALL THAT APPLY	
Medically unable	1
Not enough time	3
Problems with milk supply	8
Not publicly acceptable	2
Lack of support from the hospital or from healthcare providers	2
Lack of knowledge on breastfeeding	2
8. Please indicate your access to the following resources either now or in the past	
Access to a breast pump:	
Yes	21
No	3
Access to lactation consultant	
Yes	18
No	5
Paid maternity leave from your job	
Yes	4
No	15
Unpaid maternity leave from your job	
Yes	10
No	8
9. Who is/was your main source of support while breastfeeding? Select ALL that apply	
Father of the child	14
Grandparent to the child	10
Other family members	9
Friends/Community members	10
I did not have support	1
Additional comments about your breastfeeding experiences	
Comment 1	Don’t give up on breastfeeding. It is the best thing for your child. Good luck
Comment 2	As much as I know it is the best option for my baby it is not easy on me. It is painful and very time-consuming
Comment 3	I wished it was longer. I breastfed for 3 months
Comment 4	A lactation consultant with Healthy Start helped me
Comment 5	I felt the hospital could have been more helpful when I was there but had resources outside of the hospital I did not make it to

During the pre-registration period for the intervention, 38 respondents pre-registered for the conference using the online Eventbrite portal; of these pre-registrants, 19 individuals checked in for conference attendance, 17 completed the conference pre-test, and 15 completed the conference post-test. We report a 50% attendance rate and a 79% survey completion rate. As shown in Table 2, the minimum, maximum, and average values were calculated for pre-test and post-test data for questions 1-4. For the first four questions of the pre-test, the minimum response values were 4, 4, 4, and 2, respectively. Minimum post-test response values were 7, 7, 6, and 0, respectively. Both pre-test and post-test data sets showed maximum values of 10 for every response item. Mean pre-test values were 7.6, 7.7, 6.7, and 6.3, respectively. Mean post-test values were 8.9, 9.3, 8.4, and 8.1, respectively. Unadjusted percent changes from pre-test to post-test values for each question were calculated as 17%, 21%, 26%, and 29%, respectively. Within the handouts, participants were asked to provide feedback regarding the intervention. Questions and responses from the feedback handout are included in Table 3.

**Table 2:** Pre-test and post-test conference data collection.

Question	Pre-test, mean (range)	Post-test, mean (range)	Percent change
Knowledge about community education practices/support for breastfeeding mothers	7.6 (4–10)	8.9 (7–10)	+17%
Knowledge about other agencies and how they support breastfeeding mothers	7.7 (4–10)	9.2 (7–10)	+21%
Knowledge about developing strategies for interagency collaboration	6.7 (4–10)	8.4 (6–10)	+26%
Confidence in developing achievable goals to increase the number of breastfeeding mothers in Sarasota County	6.3 (2–10)	8.1 (0–10)	+30%

**Table 3:** Responses from conference feedback questions.

Feedback questions	
Pre-test feedback	
What are you hoping to gain from the conference?	
Comment 1	Improve collaboration and communication
Comment 2	Knowledge of ways to support moms and families through community partner services
Comment 3	Networking
Comment 4	Strategies for engaging community members in BF programs
Post-test feedback	
What did you like/enjoy about the conference?	
Comment 1	Meeting new partners/networking
Comment 2	Learning about different agencies and resources in the community
Comment 3	Speakers are local and part of community efforts. Being around like-minded people
Comment 4	Opportunities for open discussion, collaboration, and a good flow of sessions
What could have been improved?	
Comment 1	Panelists and speakers that are more representative (public and private entities, variety in race, ethnicity, and gender)
Comment 2	The date was during Spring break so it affected attendance
Comment 3	Introductions of each attendee
Comment 4	Offering a virtual option
How likely are you to attend this conference next year?	
Unlikely	0
Somewhat likely	2
Extremely likely	13

Some missing data encountered at the conference was that two individuals did not complete the post-test survey after completing the pre-test survey at the start of the conference. Additionally, fewer individuals signed into the conference compared to pre-registered attendees on Eventbrite. Unintentional problems that were faced during this project were reaching out to the target organizations for participation in the conference. Attendees were contacted through email or their organization’s website. About 50% of the individuals contacted for the conference replied to the emails or messages. Additionally, there was a lack of diversity among the conference speakers. This was mainly due to the individuals who were available for the scheduled conference, as well as the demographics of the employed individuals in the local community. Unintended costs for our intervention were providing coffee and food for conference attendees. With the help of the FDOH-Sarasota County and local food agencies, we were able to offer refreshments for conference attendees and speakers. One of the benefits that were encountered throughout the conference was the amount of embedded access we received through FDOH-Sarasota County. We had direct access to WIC, Healthy Start, and other organizations directly through the FDOH-Sarasota County. The use of their large conference rooms made it possible to accommodate all invitees.

## Discussion

Implications of breastfeeding on mother and baby

To promote breastfeeding, several key breastfeeding benefits for both mother and baby must be highlighted. Increasing evidence has suggested several short and long-term health benefits, such as decreased infection rates in infants [10]. A study performed by the World Health Organization revealed that the long-term benefits of breastfeeding reduce the risk of childhood obesity and type 2 diabetes while also demonstrating that breastfeeding was associated with higher performance on intelligence tests [10]. Regarding maternal benefits, breastfeeding has been associated with short-term benefits such as decreased postpartum blood loss and lower postpartum triglyceride and glucose levels [3]. Alternatively, evidence suggests that a shorter duration of breastfeeding is associated with a higher risk for postpartum depression [11]. Mothers must be educated on these health benefits to encourage exclusive breastfeeding further. Overall, it is readily apparent that breastfeeding has several benefits for both the mother and baby. While our quality improvement project proposes one avenue for improving breastfeeding rates at a community level, novel efforts to promote breastfeeding should be employed when considering these short-term and long-term benefits. 

Survey response considerations

The administration of the community breastfeeding survey revealed a demographic snapshot of Caucasian and Hispanic ethnicities with an average age of 31 years old. Contextualizing the age of our respondents, the reported barriers to exclusive breastfeeding aligned with our expected findings. Half of the mothers said the lack of access to paid maternity leave was a barrier, implying these new mothers continued providing financial support to their families. More so, we observed that mothers overwhelmingly expressed a goal of long-term exclusive breastfeeding and confidence in their breastfeeding knowledge. However, only two survey participants reported exclusive breastfeeding through 12 months. Subsequently, we concluded that maternal knowledge and motivation were not a deficit in promoting exclusive breastfeeding rates; instead, appropriate breastfeeding support proved to be a more fruitful aim. Therefore, our second PDSA cycle set the development of more robust community agency maternal support as a goal.

When examining the more significant trends regarding individual attendees’ knowledge of other community agencies’ missions and services offered, we recorded scores within the middle range of confidence. Pre-test question 2 addressed attendees’ understanding of the services provided to breastfeeding mothers by other community entities and showed a mean value of 7.7 out of 10. Pre-test question 3 measured individuals’ knowledge of establishing or developing a strategy to begin interagency collaboration and showed a mean value of 6.7 out of 10. Both domains improved by over 20% (21% and 26%, respectively), demonstrating that our intervention provided an effective means of interagency education. In conjunction with the S.M.A.R.T. goal writing workshop at the end of our conference, we believe this led to the percent change of test question 4 displaying the largest increase. The reported increase in collaboration confidence reflects the need for more substantial interagency infrastructure and greater emphasis on wrap-around services concerning breastfeeding support in Sarasota County. We believe this will lead to a demonstrable increase in service coordination to ensure exclusive breastfeeding at six months and beyond.

Many conference participants reported concerns that their services often overlapped with each other’s patient/client populations. Supporting interagency knowledge within this newly identified domain could provide another avenue to increase the number of exclusively breastfeeding mothers at six months and beyond. It is important to note that the conference or other mechanisms for interagency collaboration developed in this project have not been otherwise cited in the literature as an intervention to increase exclusive breastfeeding practices. To our knowledge, our intervention is the only currently active interagency resource for breastfeeding providers in Sarasota County. Further, this is the only dynamic platform for sharing breastfeeding service strategies between hospital breastfeeding support departments and community breastfeeding partners.

We learned from our open-ended feedback questions that participants found value in the conference due to the scarcity of chances to network with other breastfeeding providers in the community. It was further learned that the community encouraged broadening the definition of what constitutes a support provider within the realm of breastfeeding. Lactation specialists welcomed the involvement of doulas, midwives, and other healthcare professionals exposed to all phases of pregnancy. Looking forward, we recommend expanding the outreach pool for the next conference to reach different communities and broaden the demographics of conference attendees and speakers.

Limitations

Limitations of the Maternal Breastfeeding Community Survey included a small sample size and limited demographic data collection (i.e., zip code, area of residence, etc.) of respondents. Additionally, variations in how the survey was administered posed a limitation.

Regarding the conference, we observed a 50% lower in-person attendance rate than online registration. We suspect that considerations of holding the meeting during a weekday morning that coincided with Sarasota County Schools’ spring break likely proved to be an obstacle to attendance. Reliance on a locale provided by community partners could pose a challenge in the annual execution of this conference. Selecting the FDOH-Sarasota County main campus as a host site provided a location for which most participants were already familiar. However, this created a transportation limitation due to its northern location in Sarasota County’s large vertical geography.

Future considerations

Looking at existing literature, a considerable amount of data was available regarding individual community initiatives that worked directly with breastfeeding mothers to increase exclusive breastfeeding rates. As evidenced by the survey feedback, interagency collaboration does offer a promising means to increase breastfeeding rates. While there is an understanding of the significant benefits of breastfeeding based on the efforts in basic, clinical, and social sciences, there is still a need for continued improvement in collaborative research efforts among multiple disciplines [12]. Based on the findings of Azad et al., recommendations to broaden the utilization of alternative modes of breast milk, including donor breast milk, may offer a profound initiative to increase breastfeeding rates. Aligning with the findings of our quality improvement project, improving awareness of breastfeeding benefits and challenges for communities may also be helpful [12].

Regarding other methods of improving breastfeeding rates, a recent meta-analysis found that peer counseling was an effective strategy associated with higher breastfeeding rates among young mothers [13]. Studies that used more than one intervention (i.e., peer support, telephone calls, home nursing care) had higher success rates than those that used only one intervention method [13]. Using a three-phase intervention, another study recorded an increase in breastfeeding rates from 2.4% to 49% among breastfeeding mothers who did not use supplemental formula or non-breastmilk liquids during their hospital stay [14]. Additionally, increasing the involvement of obstetrician-gynecologists may be beneficial in enhancing breastfeeding practices at the levels of public policy and clinical practice [6]. These findings suggest alternative avenues for community involvement and collaboration for future studies and interventions.

While we concluded that maternal knowledge and motivation did not serve as limitations in increasing breastfeeding rates, appropriate breastfeeding support was a more realistic target. Additional considerations for information that the breastfeeding survey could elicit would be awareness of the health benefits of breastfeeding, the mother’s area of work, and the availability of space for breastfeeding at work. A few studies have shown that including fathers in breastfeeding education is potentially important. An increase in breastfeeding rates by the end of the third and sixth month was recorded among families with increased knowledge by both the mother and father [15]. While individual knowledge rates increased using informational interventions, understanding of breastfeeding from both parents caused a significant increase in breastfeeding frequency [15]. Targeting familial support systems may be an additional route for future studies.

## Conclusions

Given the numerous benefits of breastfeeding for both the mother and infant, along with the current breastfeeding rates in the United States, increasing the number of breastfeeding mothers was the primary focus of our intervention. While there are several factors contributing to the current rates of breastfeeding, there is an apparent need for increased communication and goal-setting among community agencies involved in breastfeeding initiatives that can ultimately increase the number of newborns being breastfed at six months of life and beyond. Following the completion of our intervention, it is evident that there is a need for improvement in the pipeline of expanding awareness and collaboration among community agencies that share the common goal of increasing the number of breastfeeding mothers in Sarasota County.

Future iterations of this conference can be aimed at increasing outreach to allow for sustainable collaboration. It may also be beneficial to continually assess necessary improvements to the conference as needed. Limitations related to our first PDSA cycle include different initiation times for survey administration among community agencies, small sample sizes, and limited demographic data collection (i.e., zip code, area of residence, etc.) of respondents. Additional limitations related to our second PDSA cycle include time constraints to work on the project, a relatively low number of conference attendees, limited diversity among conference speakers, and attrition in our post-test administration.

## Figures and Tables

**Figure 6:**
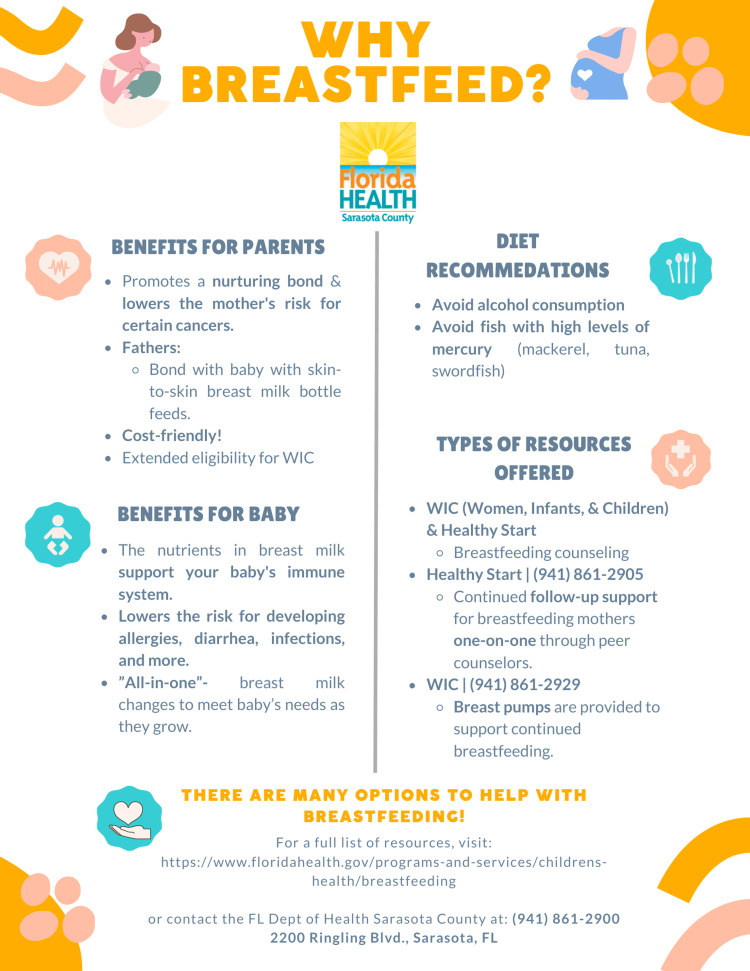
Breastfeeding educational flyer distributed at the 2023 Newtown Community Screenings & Health Fair.

**Figure 7:**
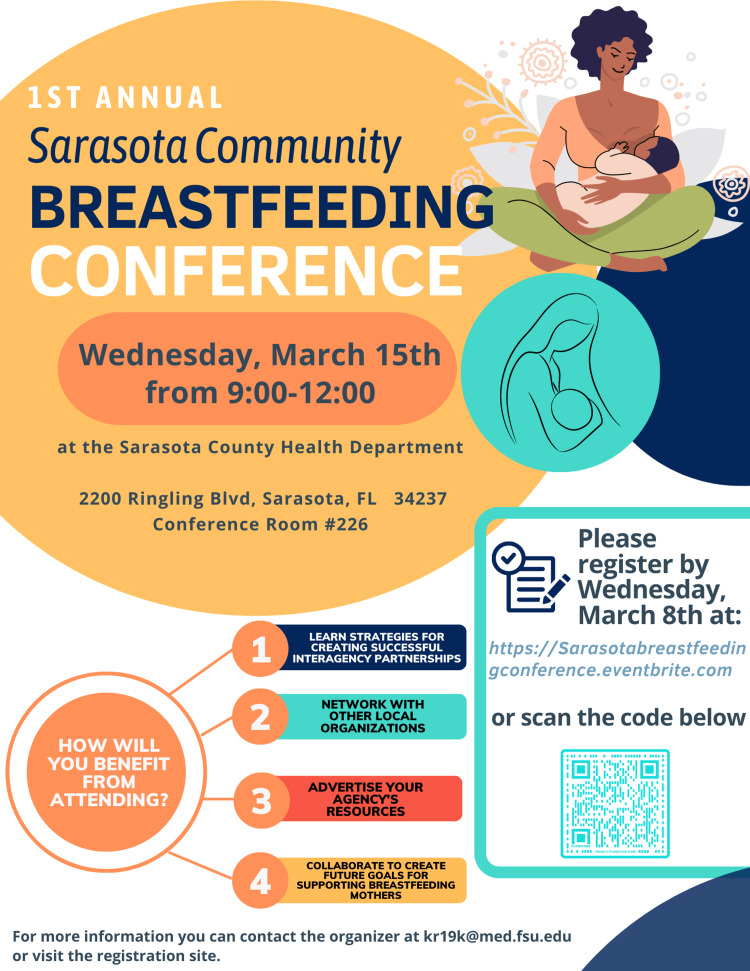
Inaugural Sarasota Community Breastfeeding Conference advertisement flyer.

**Figure 8:**
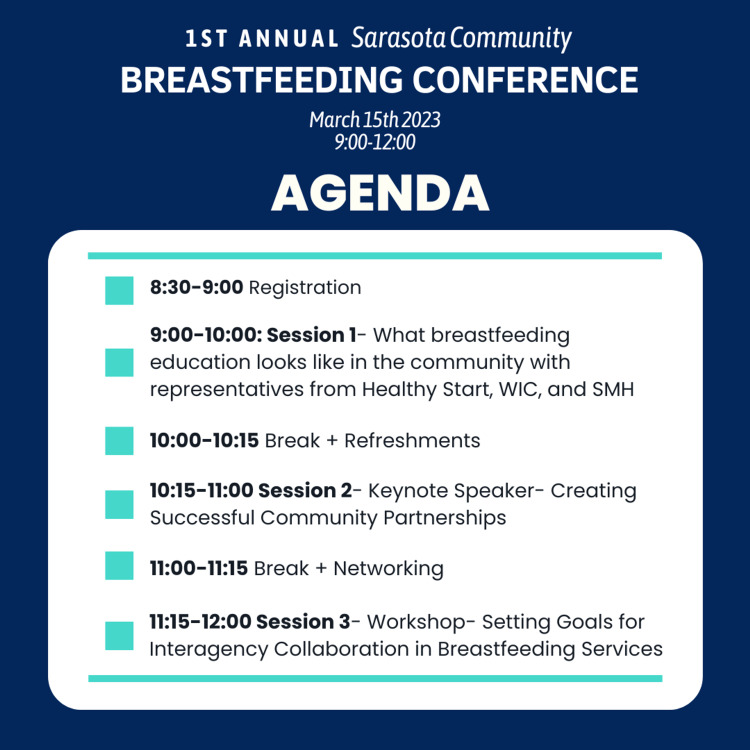
Inaugural Sarasota Community Breastfeeding Conference Agenda.

**Figure 9:**
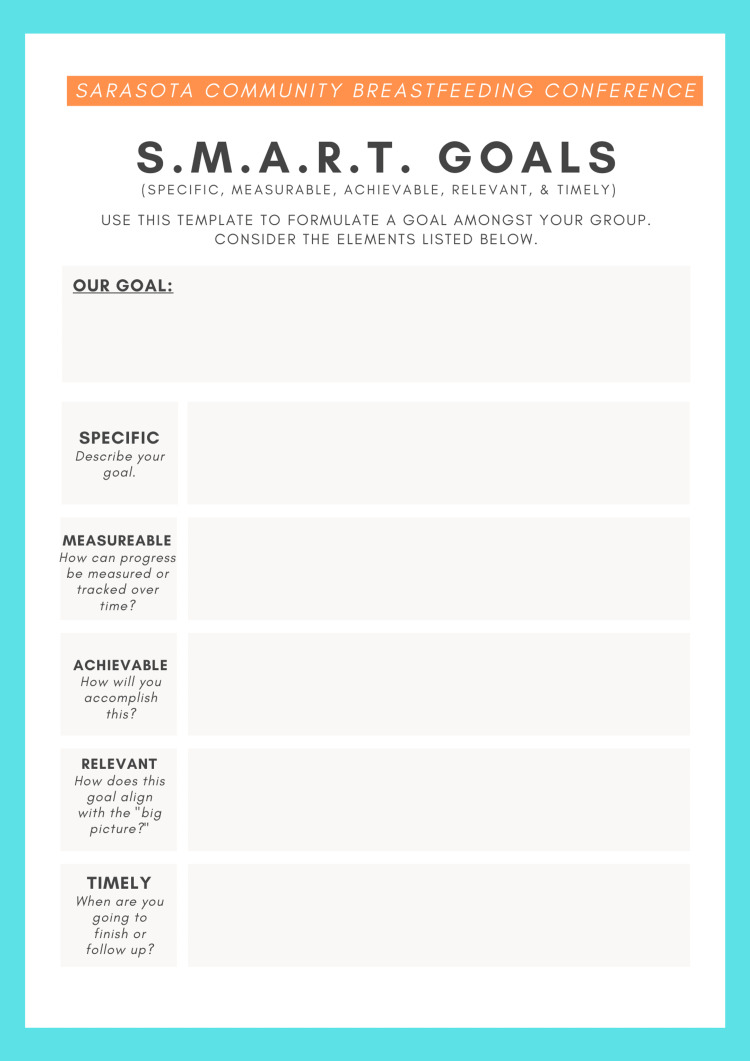
Specific, Measurable, Achievable, Realistic, and Timely (S.M.A.R.T.) goals handout provided to conference participants.

## Appendices

Appendix 1

Appendix 2

Appendix 3

Appendix 4
